# Guillain-Barré Syndrome Following Orthopedic Trauma and Surgical Fixation: A Case of Acute Inflammatory Demyelinating Polyradiculoneuropathy

**DOI:** 10.7759/cureus.112323

**Published:** 2026-07-09

**Authors:** Ajinkya Kakade, Shilpa Deoke, Sayali D Kolse, Fahad Idrees Shaikh, Nishka Tiwari

**Affiliations:** 1 General Medicine, N.K.P. Salve Institute of Medical Sciences and Research Centre and Lata Mangeshkar Hospital, Nagpur, IND; 2 Internal Medicine, N.K.P. Salve Institute of Medical Sciences and Research Centre and Lata Mangeshkar Hospital, Nagpur, IND; 3 Medical Education, N.K.P. Salve Institute of Medical Sciences and Research Centre and Lata Mangeshkar Hospital, Nagpur, IND

**Keywords:** acute inflammatory demyelinating polyradiculoneuropathy, demyelinating neuropathy, guillain-barré syndrome, ivig, orthopedic surgery, postsurgical guillain-barré syndrome

## Abstract

Guillain-Barré syndrome (GBS) is an acute inflammatory immune-mediated polyradiculoneuropathy characterized by rapidly progressive ascending weakness, generalized areflexia, and varying degrees of cranial nerve involvement. Although antecedent respiratory and gastrointestinal infections are the most commonly seen triggers, an association between surgery and GBS has increasingly been recorded in recent years. Recognition in postoperative patients may be delayed because neurological deficits are commonly attributed to postoperative complications, prolonged immobility, metabolic abnormalities, or medication-related adverse effects. Although GBS following orthopedic surgery has been increasingly reported in recent years, it remains an important diagnostic challenge because postoperative neurological deficits are frequently attributed to surgical, metabolic, or medication-related causes, resulting in delayed diagnosis and treatment. We report the case of a 19-year-old male who developed rapidly progressive ascending quadriparesis with areflexia and right lower motor neuron facial palsy around 10 days after open reduction and internal fixation for left tibia and fibula fractures. Nerve conduction studies demonstrated demyelinating sensorimotor polyradiculoneuropathy consistent with the acute inflammatory demyelinating polyradiculoneuropathy type of GBS. Cerebrospinal fluid analysis showed albuminocytological dissociation. The patient was treated with intravenous immunoglobulin therapy and demonstrated remarkable neurological recovery with near-complete restoration of motor function at follow-up. This case highlights the diagnostic challenges associated with postoperative GBS and emphasizes the importance of maintaining a high index of suspicion in patients presenting with rapidly progressive weakness after orthopedic procedures. Early electrophysiological evaluation and prompt immunotherapy can result in excellent neurological recovery.

## Introduction

Guillain-Barré syndrome (GBS) is an acute immune-mediated neuropathy affecting peripheral nerves and spinal nerve roots, commonly presenting with rapidly progressive symmetrical weakness, generalized hyporeflexia or areflexia, and varying degrees of sensory, cranial nerve, and autonomic involvement [1]. The global incidence of GBS has been estimated to range from 1 to 2 cases per 100,000 population annually, with acute inflammatory demyelinating polyradiculoneuropathy (AIDP) representing the most prevalent subtype worldwide [2]. Antecedent respiratory or gastrointestinal infections are identified as triggers in the majority of patients, with *Campylobacter jejuni*, cytomegalovirus, Epstein-Barr virus, and *Mycoplasma pneumoniae* often implicated as pathogens [3]. Trauma and surgical procedures have increasingly been reported in temporal association with GBS, although a definitive causal relationship has not been established. GBS has been described following orthopedic, gastrointestinal, cardiac, spinal, and neurosurgical procedures [4]. Despite increasing recognition, diagnosis remains challenging because early neurological symptoms may mimic postoperative pain, immobilization, electrolyte abnormalities, medication-related adverse effects, or traumatic nerve injury. Consequently, timely diagnosis and treatment may be delayed. Recognition of GBS temporally being associated following orthopedic surgery remains clinically challenging because early neurological symptoms may initially be attributed to prolonged immobilization, postoperative pain, electrolyte abnormalities, medication effects, or traumatic neurological injury [5,6]. The mechanisms underlying GBS occurring after surgery remain incompletely understood. Proposed mechanisms include perioperative immune dysregulation, cytokine-mediated inflammatory activation, and exposure of neural antigens leading to autoimmune demyelination [7].

We report a case of acute inflammatory demyelinating polyradiculoneuropathy (AIDP) developing after open reduction and internal fixation of tibia and fibula fractures. This case highlights the importance of recognizing GBS in the differential diagnosis of new-onset postoperative weakness, particularly when accompanied by generalized areflexia and cranial nerve involvement.

## Case presentation

A previously healthy 19-year-old male presented with progressive weakness involving all four limbs (quadriparesis) for four days. The weakness initially affected the lower extremities and gradually progressed to involve both upper limbs. The progression was ascending, symmetrical, and predominantly proximal. Initially, the patient experienced difficulty walking and climbing stairs, which progressed to requiring assistance to rise from a seated position. Subsequently, he developed difficulty performing fine motor activities such as buttoning his shirt and combing his hair. Three days after the onset of limb weakness, the patient developed facial asymmetry associated with slurring of speech and dysphagia. Difficulty swallowing initially involved solids and later progressed to liquids. There was no history of fever, diarrhea, upper respiratory tract infection, recent vaccination, visual disturbances, sensory loss, bowel or bladder dysfunction, seizures, altered sensorium, or loss of consciousness. The patient sustained a road traffic accident resulting in fractures of the left tibia and fibula and underwent open reduction and internal fixation the following day at an outside hospital. He presented to our institution on approximately postoperative day 14, after developing progressive neurological symptoms beginning around postoperative days 10-11. The immediate postoperative period was uneventful, and the patient was mobilizing with support before the onset of neurological symptoms. Weakness developed approximately 10-11 days after the surgery.

The patient had no known history of diabetes mellitus, hypertension, autoimmune disease, thyroid disorder, or any other chronic medical illness. He was not receiving any long-term medications and had no history of drug allergies. There was no family history of autoimmune or neurological disorders. He was a smoker but did not consume alcohol or recreational drugs. There was no history of recent vaccination, COVID-19 infection, upper respiratory tract infection, gastrointestinal illness, or any other identifiable antecedent trigger preceding the onset of neurological symptoms. The patient’s body mass index was 17.8 kg/m².

General physical examination was unremarkable with no evidence of pallor, icterus, cyanosis, clubbing, edema, lymphadenopathy, or cutaneous lesions. On examination, the patient was alert, cooperative, and well oriented to time, place, and person. Vital parameters were stable. Higher mental functions were intact.

Cranial nerve examination demonstrated right lower motor neuron facial nerve palsy characterized by loss of forehead wrinkling, incomplete closure of the right eye, flattening of the right nasolabial fold, and deviation of the angle of the mouth to the left. Bulbar involvement was evident as dysarthria and dysphagia.

Motor examination revealed hypotonia involving all four limbs. Muscle power, according to the Medical Research Council grading system [8], was 4/5 in bilateral upper limbs and 1/5 in bilateral lower limbs. Weakness was symmetrical, predominantly affecting the lower limbs, consistent with an ascending pattern. Deep tendon reflexes were absent bilaterally in both upper and lower limbs. Plantar responses were not elicitable. The weakness was symmetrical and predominantly involved the lower limbs, with greater severity in the proximal muscle groups and relative sparing of the upper limbs at presentation. This pattern of ascending weakness, characterized by more severe lower limb involvement with subsequent progression to the upper limbs, is typical of GBS and further supported the clinical diagnosis.

Sensory examination, including pain, touch, temperature, vibration, and proprioception, was normal. Cerebellar examination revealed no abnormalities. Gait could not be assessed due to severe lower limb weakness. Respiratory examination showed no evidence of accessory muscle use or paradoxical breathing. There were no clinical features of autonomic instability at presentation. The clinical presentation of rapidly progressive ascending weakness with generalized areflexia strongly suggested GBS.

Nerve conduction studies (NCS) were subsequently performed in the bilateral median, ulnar, left tibial, left peroneal, and sural nerves. Motor conduction parameters of the bilateral median nerves were within normal limits, including preserved F-wave responses. Motor NCS demonstrated preserved distal latency, compound muscle action potential (CMAP) amplitudes, and conduction velocities in both median nerves. In contrast, bilateral ulnar nerves as well as the left tibial and left peroneal nerves showed prolonged distal latencies, reduced CMAP amplitudes, and decreased conduction velocities. Conduction block was observed in both ulnar nerves.

Sensory NCS demonstrated preserved amplitudes and peak latencies in bilateral median and ulnar sensory nerves, although sensory conduction velocities were reduced. The left sural nerve showed normal latency, amplitude, and conduction velocity. F-wave responses were absent in most tested nerves except for the bilateral median nerves.

Motor conduction parameters of the bilateral median nerves were within normal limits, including preserved F-wave responses. This relative sparing of the median nerves, in contrast to the abnormalities observed in the ulnar, tibial, and peroneal nerves, likely reflects the patchy and non-uniform distribution of demyelination that may occur in the early stages of AIDP.

The electrophysiological findings fulfilled the Hadden et al. [9] electrodiagnostic criteria for AIDP, based on the presence of prolonged distal motor latencies, reduced motor conduction velocities, conduction block, and abnormal F-wave responses involving multiple motor nerves. Detailed nerve conduction study findings are summarized in Table 1.

**Table 1:** Nerve conduction study findings. CMAP = compound muscle action potential; SNAP = sensory nerve action potential

Nerve/Study	Electrophysiological findings
Bilateral median motor nerves	Normal distal latency, CMAP amplitude, and conduction velocity
Bilateral ulnar motor nerves	Increased distal latency, reduced CMAP amplitude, decreased conduction velocity, and conduction block
Left tibial motor nerve	Increased latency, reduced amplitude, and decreased conduction velocity
Left peroneal motor nerve	Increased latency, reduced amplitude, and decreased conduction velocity
Bilateral median and ulnar sensory nerves	Normal SNAP amplitudes and peak latencies with reduced sensory conduction velocities
Left sural sensory nerve	Normal latency, amplitude, and conduction velocity
F-wave study	F-wave responses absent in most tested nerves except the bilateral median nerves
Overall interpretation	Findings suggestive of demyelinating sensorimotor polyradiculoneuropathy involving all four limbs, consistent with acute inflammatory demyelinating polyradiculoneuropathy variant of Guillain–Barré syndrome

Table 1 demonstrates features of demyelination, including delayed distal latencies, conduction block, reduced conduction velocities, and absent F-wave responses suggestive of demyelinating neuropathy. Cerebrospinal fluid (CSF) analysis demonstrated albumniocytological dissociation, further supporting the diagnosis of GBS. CSF findings are summarized in Table 2.

**Table 2:** CSF analysis findings. CSF = cerebrospinal fluid

Parameter	Findings	Reference range
Appearance	Clear and colorless	-
Total leukocyte count	3 cells/mm³	0–5 cells/mm^3^
CSF protein	190 mg/dL	15–45 mg/dL
CSF glucose	58 mg/dL	50–80 mg/dL
Gram stain/Culture	No organisms detected	No organisms detected
CSF impression	Albuminocytological dissociation suggestive of Guillain-Barré syndrome	-

Routine hematological and biochemical investigations were within normal limits. The laboratory investigations are summarized in Table 3.

**Table 3:** Laboratory investigations including hematological and biochemical investigations. AST - aspartate aminotransferase; SGOT = serum glutamate-oxaloacetic transaminase; ALT = alanine aminotransferase; SGPT = serum glutamate-pyruvate transaminase; INR = international normalized ratio; HIV = human immunodeficiency virus; HBsAg = hepatitis B surface antigen; HCV = hepatitis C virus

Parameter	Patient value	Reference range
Hemoglobin	13.8 g/dL	13.0–17.0 g/dL
Total leukocyte count	7,800 cells/mm³	4,000–11,000 cells/mm³
Differential leukocyte count		
Neutrophils	80%	40–75%
Lymphocytes	15%	20–45%
Eosinophils	3%	1–6%
Monocytes	2%	2–10%
Platelet count	2.5 × 10⁵/mm³	1.5–4.5 × 10⁵/mm³
Erythrocyte sedimentation rate	12 mm/hour	0–20 mm/hour
C-reactive protein	<5 mg/L	<5 mg/L
Random blood glucose	96 mg/dL	70–140 mg/dL
Blood urea	28 mg/dL	15–40 mg/dL
Serum creatinine	0.8 mg/dL	0.6–1.3 mg/dL
Serum sodium	139 mEq/L	135–145 mEq/L
Serum potassium	4.2 mEq/L	3.5–5.0 mEq/L
Serum chloride	102 mEq/L	98–106 mEq/L
Serum calcium	9.1 mg/dL	8.5–10.5 mg/dL
Serum magnesium	2.0 mg/dL	1.7–2.4 mg/dL
Total bilirubin	0.7 mg/dL	0.2–1.2 mg/dL
Direct bilirubin	0.2 mg/dL	0–0.3 mg/dL
AST (SGOT)	28 U/L	10–40 U/L
ALT (SGPT)	25 U/L	7–56 U/L
Alkaline phosphatase	92 U/L	44–147 U/L
Total protein	7.1 g/dL	6.0–8.3 g/dL
Serum albumin	4.2 g/dL	3.5–5.0 g/dL
Prothrombin time	12.5 seconds	11–14 seconds
INR	1.0	0.8–1.2
Activated partial thromboplastin time	31 seconds	25–35 seconds
HIV 1 & 2 serology	Non-reactive	Non-reactive
HBsAg	Negative	Negative
Anti-HCV antibody	Negative	Negative
Urine routine examination	Normal	Normal

The patient was admitted to the intensive care unit for close monitoring because of the risk of respiratory muscle involvement and autonomic dysfunction. Throughout hospitalization, he maintained normal oxygen saturation on room air, showed no clinical evidence of respiratory compromise, and did not require supplemental oxygen, non-invasive ventilation, or mechanical ventilation. Intravenous immunoglobulin (IVIG) was administered at the standard recommended dose of 2 g/kg body weight, divided over five consecutive days (0.4 g/kg/day), along with supportive care and physiotherapy. Following therapy, the patient demonstrated gradual neurological improvement. Lower limb strength improved progressively, cranial nerve deficits resolved, and the patient regained the ability to ambulate independently. Follow-up evaluation after approximately one month revealed complete neurological recovery with restoration of muscle power to 5/5 in all four limbs. The chronological sequence of clinical events and management is summarized in Table 4.

**Table 4:** Timeline of clinical progression and management. NCS = nerve conduction studies; CSF = cerebrospinal fluid; AIDP = acute inflammatory demyelinating polyradiculoneuropathy; GBS = Guillain-Barré syndrome; IVIG = intravenous immunoglobulin

Timeline	Clinical event
Day 0	Road traffic accident resulting in a fracture of the left tibia and fibula
Day 1	Underwent open reduction and internal fixation at an outside hospital
Postoperative period	Uneventful recovery; patient mobilizing with support
Postoperative day 10–11	Onset of progressive weakness in both lower limbs
Postoperative day 11–12	Weakness progressed to involve both upper limbs
Postoperative day 13	Difficulty walking, rising from a sitting position, and performing fine motor activities
Postoperative day 13–14	Development of facial asymmetry, dysarthria, and dysphagia
Postoperative day 14	Presented to our institution; neurological examination revealed hypotonia, generalized areflexia, quadriparesis, and right lower motor neuron facial nerve palsy
Postoperative day 14	NCS and CSF findings consistent with the AIDP variant of GBS
Postoperative day 15	Patient admitted to the intensive care unit for monitoring
Postoperative day 15–19	IVIG administered at 20 g/day for 5 days
During hospitalization	Gradual improvement in limb strength and cranial nerve deficits
One-month follow-up	Complete neurological recovery with restoration of motor power to 5/5 in all four limbs

## Discussion

GBS following orthopedic surgery is an increasingly recognized neurological complication following a wide range of surgical procedures, including orthopedic operations such as fracture fixation, arthroplasty, and spinal surgery. Rather than its rarity, the major clinical challenge lies in its early recognition because postoperative weakness is often initially attributed to expected surgical recovery, pain, prolonged immobilization, medication effects, electrolyte disturbances, or traumatic nerve injury [10].

Recent literature suggests that surgery is increasingly recognized as a potential independent trigger for GBS, although the exact incidence remains uncertain because most available evidence consists of retrospective studies, case series, and individual case reports. Postsurgical GBS has been described following orthopedic, gastrointestinal, cardiac, spinal, and neurosurgical procedures, indicating that surgical stress itself may contribute to disease onset in susceptible individuals. The proposed mechanisms include perioperative immune dysregulation, cytokine-mediated inflammatory activation, transient immunosuppression followed by immune rebound, and exposure of neural antigens secondary to tissue injury. Orthopedic trauma and fracture fixation may further augment these immune responses because of extensive tissue damage and the inflammatory cascade associated with bone injury and surgical manipulation.

The educational value of the present case lies in demonstrating how the characteristic clinical pattern of rapidly progressive ascending weakness, generalized areflexia, bulbar symptoms, and cranial nerve involvement, together with supportive electrophysiological and CSF findings, facilitated early diagnosis of GBS. Prompt recognition allowed the timely initiation of IVIG therapy before respiratory failure developed, resulting in complete neurological recovery. This case reinforces the importance of considering GBS whenever progressive neurological deficits occur after otherwise uncomplicated orthopedic surgery. Delayed detection in postoperative patients remains an important clinical concern as early symptoms may mimic postoperative fatigue, pain-related immobility, electrolyte disturbances, drug-related effects, or traumatic neurological injury [11].

The present case revealed diagnostic symptoms of the AIDP variant, including ascending symmetrical weakness, generalized areflexia, cranial nerve involvement, bulbar dysfunction, and demyelinating abnormalities on nerve conduction investigations. The temporal association between surgery and the onset of neurological symptoms significantly supported the diagnosis of GBS following orthopedic surgery [12]. Although the exact mechanism producing GBS following surgery remains unknown, perioperative immune activation and inflammatory dysregulation are considered important contributors. Surgical stress may trigger abnormal immune responses through cytokine release and exposure of neural antigens, ultimately leading to autoimmune-mediated peripheral nerve demyelination [13]. Electrophysiological evaluation plays a key role in establishing the diagnosis and differentiating demyelinating from axonal types of GBS. Typical findings in AIDP include prolonged distal latencies, conduction block, reduced conduction velocities, temporal dispersion, and absent or prolonged F-wave responses, all of which were seen in the present patient [14]. CSF analysis demonstrating albuminocytological dissociation further supported the diagnosis. However, normal CSF findings may occasionally be seen during the early stages of disease, emphasizing the importance of correlating clinical and electrophysiological findings [15]. Early recognition of GBS is essential because rapid progression may lead to respiratory insufficiency, autonomic instability, and life-threatening complications requiring intensive care monitoring. Previous studies have reported that a significant proportion of patients eventually require ventilatory support during the disease course [16]. IVIG and plasmapheresis remain the standard first-line therapies for GBS and have been shown to improve neurological outcomes when initiated early in the disease course. Our patient demonstrated an excellent response to IVIG therapy with near-complete neurological recovery, highlighting the importance of timely diagnosis and prompt immunotherapy [16].

In the postoperative setting, the differential diagnosis of acute weakness includes postoperative peripheral nerve injury, spinal cord pathology, electrolyte disturbances, critical illness neuropathy, medication-related neuromuscular weakness, and GBS. In the present case, postoperative nerve injury was considered unlikely because the patient developed symmetrical ascending weakness involving all four limbs, generalized areflexia, and cranial nerve involvement, which cannot be explained by a focal surgical nerve injury following tibia and fibula fixation. Furthermore, NCS demonstrated a generalized demyelinating sensorimotor polyradiculoneuropathy rather than a localized peripheral nerve lesion, and CSF analysis showed albuminocytological dissociation, strongly supporting the diagnosis of AIDP.

The prognosis of GBS following orthopedic surgery is largely determined by the severity of neurological involvement and the timeliness of diagnosis and treatment. Although some patients require ventilatory support and prolonged rehabilitation, early recognition and prompt initiation of IVIG or plasmapheresis are associated with favorable neurological recovery. In the present case, early diagnosis and treatment resulted in complete functional recovery at follow-up.

## Conclusions

GBS following orthopedic surgery is an important diagnostic consideration in patients presenting with acute ascending weakness following orthopedic trauma or surgery. A high index of suspicion is essential, as the clinical presentation may mimic other postoperative complications, including peripheral nerve injury, metabolic disturbances, or medication-related weakness. Careful consideration of the differential diagnosis, together with early electrophysiological evaluation and CSF analysis, facilitates timely diagnosis. Close respiratory monitoring, prompt initiation of immunotherapy, and multidisciplinary management involving neurologists, intensivists, rehabilitation specialists, and orthopedic surgeons are essential to optimize neurological recovery and prevent life-threatening complications.
